# Generation of green electricity from sludge using photo-stimulated bacterial consortium as a sustainable technology

**DOI:** 10.1186/s12934-023-02187-y

**Published:** 2023-09-15

**Authors:** Amal S. Othman, Nashwa A. Ahmed, Mona S. Elneklawi, Mansour M. Hassan, Mahmoud Abd El-Mongy

**Affiliations:** 1 Faculty of Applied Health Sciences Technology, October 6 University, P.O. Box 12585, El- Giza, Egypt; 2 Microbial Biotechnology Department, Genetic Engineering and Biotechnology Institute, Sadat City University, Sadat city, Egypt

**Keywords:** Electrogenic Bacteria, Microbial Fuel Cell (MFC), Green Electricity, Sludge, pH, Temperature, photo-stimulation

## Abstract

Microbial fuel cell (MFC) is a bio-electrical energy generator that uses respiring microbes to transform organic matter present in sludge into electrical energy. The primary goal of this work was to introduce a new approach to the green electricity generation technology. In this context a total of 6 bacterial isolates were recovered from sludge samples collected from El-Sheikh Zayed water purification plant, Egypt, and screened for their electrogenic potential. The most promising isolates were identified according to 16S rRNA sequencing as *Escherichia coli* and *Enterobacter cloacae*, promising results were achieved on using them in consortium at optimized values of pH (7.5), temperature (30°C) and substrate (glucose/pyruvate 1%). Low level red laser (λ = 632.8nm, 8mW) was utilized to promote the electrogenic efficiency of the bacterial consortium, maximum growth was attained at 210 sec exposure interval. In an application of adding standard inoculum (10^7^ cfu/mL) of the photo-stimulated bacterial consortium to sludge based MFC a significant increase in the output potential difference values were recorded, the electricity generation was maintained by regular supply of external substrate. These results demonstrate the future development of the dual role of MFCs in renewable energy production and sludge recycling.

**Supplementary Information:**

The online version contains supplementary material available at 10.1186/s12934-023-02187-y.

## Introduction

Energy requirement is ever increasing throughout the world, electricity is considered the basic energy in our daily life. Seeking alternative cheap and ecofriendly sources of energy generation have become a must [1]. Microbial fuel cell (MFC) is considered a bio-electrical energy system that converts chemical energy contained in organic substrates into electrical energy by the microbial activities, so it acts as a promising sustainable inexpensive alternative for renewable energy generation [2], the efficiency of MFC technology comes from the capability of electrogenic bacteria to oxidize organic matter under anaerobic conditions [3], the oxidation process produces electrons and protons, the electrons released at the anode travel through an external electrical circuit to the cathode, creating a flow of electric current, while the protons move directly to the cathode through the solution [4]. Studies reported that transferring electrons to the anode can take place either by mediator electron transfer via an exogenous redox mediator [5] or by direct electron transfer as the microbe physically contacted to the anode forming a biofilm [6]. Traditional wastewater treatment methods faced significant difficulties from high operational costs, high energy usage, and environmental pollution [7]. In addition to green energy production electrogenic microbes have enzymatic potential to degrade different macromolecules with high conversion efficiency compared to other electrochemical cells [8] Biodegradable substrates are considered as electron sources in the MFC, these substrates range from simple compounds to complex organic compounds [9], previous reports documented that sludge is used as a fuel for several anaerobic and facultative anaerobic bacterial species because of its nutrient richness and year-round availability. It is made up of effluents obtained from urban, industrial, and other sources, which are regarded as the primary source of energy to produce electricity [10]. Pure or mixed cultures of microorganisms can be used in MFC, however mixed cultures are more efficient energy generators [11]. Electrogenic microorganisms are obtained from widely used sources such as soil or marine sediment, the natural microbial population, and brewery wastewater are added to mixed cultures to increase biological constancy [12]. Suitable environmental conditions including pH, temperature and nutrients are required for the growth and multiplication of electrogenic bacteria [13]. Temperature and pH affect the activity of enzymes, which will have a great impact on the growth and reproduction of microorganisms. When the temperature is greater or lower than the optimum value, the intracellular enzymatic activity will decrease, thus affecting the power generation potentiality of the MFC [14], also environmental pH is closely related to the life activities and metabolic performance of electrogenic bacteria, so the power generation capacity of MFCs varies greatly under different pH conditions [15]. In addition, studies have reported that the lack or excess of nutrients in the growth environment of microorganisms will directly affect their metabolic activities consequently their electricity production [16], Kim et al [17] reported that the produced power was more efficient on using glucose compared to other substrates. Light biotechnology is a recent field that contributes to the studies related to the environment through the photo stimulation of the bioenergetic processes of microorganisms [18]. Photo-energizing systems include low level red laser that emits light with a relatively narrow spectral band at a certain wavelength [19], it is hypothesized that induction of the photo-modulatory microbial system by red laser promotes the increase in cellular biomass and protein synthesis [20], since this inducer tool is cost -effective so it is introduced in many environmental and medical applications.

The aim of the present work is to isolate, screen and identify the most potent electrogenic bacterial isolates from sludge samples, assess the effect of different environmental factors and different substrates on their growth, finally investigate the effect of photo-stimulation using low level red laser on the electrogenic potentiality of the bacterial consortium to introduce a new approach in boosting the efficiency of green electricity production.

## Material and methods

### Materials

All materials, chemicals and reagents are of analytical grade and were purchased from Sigma Aldrich.

### Sample collection

Sludge samples were collected throughout the year 2021 from El-Sheikh Zayed water purification plant, Giza, Egypt. Triplicate samples were collected in sterile one-liter bottles in January (S1), April (S2), July (S3) and October (S4), the samples were transported to the laboratory in a cooling box, where they immediately stored in a refrigerator at 4°C until processing within 24 h, different Physicochemical parameters had been carried out using inductively coupled plasma/Mass spectrometry (ICP/MS) (Thermo. Fisher, USA).

### Enumeration of total bacterial counts

For quantitative analysis, sludge samples were vortexed for 3 mins to be completely homogenous, one mL of each sample was suspended separately in 9 mL of sterile saline, a series of decimal dilutions were prepared, 0.1 mL of each sample was plated on nutrient agar medium and incubated for 24 h at 37°C, total viable count (TVC) was expressed as colony forming units (cfu/mL) of the sample, experiments were undertaken in triplicates [21].

No. of cfu/mL = No. of colonies counted × Dilution factor /Volume of sample taken

### Construction of the microbial fuel cell (MFC) system

**Fig. 1:**
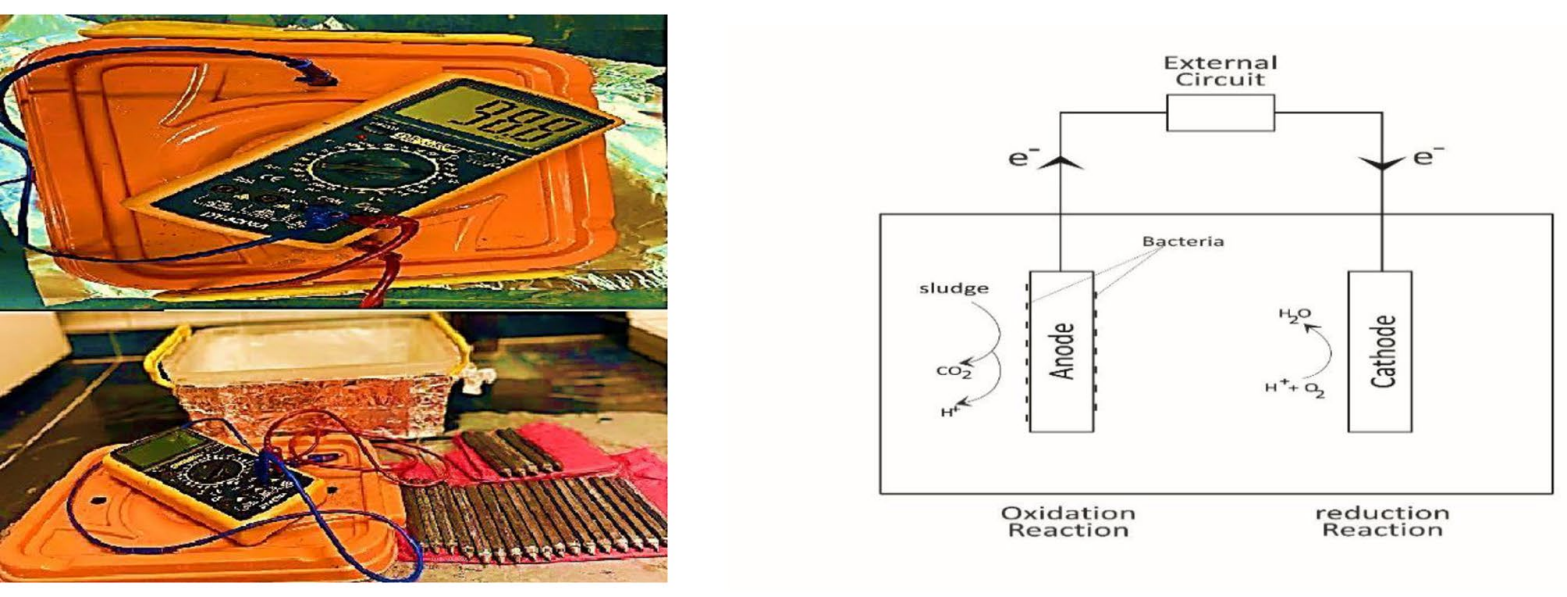
Schematic diagram of the microbial fuel cell

Single chambered Microbial fuel cell (MFC) was implemented in a rectangular plastic chamber with an inner working volume of one liter to suit the amount of the sample and the size of the electrodes used. The MFC was equipped with two graphite rods to serve as the two electrodes (anode and cathode) with an active area 25cm^2^. A positive heavy load clamp wire was used to be connected to the anode and another negative one is connected to the cathode (The cathode electrode was coated with Poly tetrafluoroethylene (PTFE) diffusion layers on the air-exposed side; 60% w/v, dispersion in water). These two electrodes were placed on opposing sides and were connected to a digital multi-meter (model number AVO DT-9205A) manufactured by SUNDER Electronics, India (Fig. 1) through an external circuit across a resistance of 500 Ω. The digital multi-meter is assigned to measure the Potential difference (mV) produced by the MFCs [22].

#### Measurement of the electric potential in the collected sludge samples

The constructed MFCs were fed with 500 mL of each sludge sample diluted with phosphate buffer solution (PBS) at the ratio of 4:1, the experiments were conducted at constant temperature the digital multi-meter’s two wires were secured to the cathode and anode poles, and the readings were recorded along ten days of operation at regular intervals [23].

### Isolation, purification, and primary identification of the isolated bacterial strains

Morphologically distinct colonies were picked, isolates were purified by streaking on nutrient agar, pure cultures were maintained at 4ºC, different bacterial isolates were identified using the VITEK® MS (bioMérieux, Marcy-l’Etoile, France), an automated microbial identification system using matrix-assisted laser desorption/ionization time-of-flight mass spectrometry (MALDI-TOF-MS) and the biochemical-based VITEK®2 system (bioMérieux, Marcy-l’Etoile, France) [24].

### Screening of the most potent electrogenic bacterial isolates

For the selection of the most potent electrogenic bacterial isolates, the constructed MFCs were sterilized via rinsing with 70% Ethanol and UV irradiated for 30 min, they were seeded with the prepared basal medium (BM) using the following constituents (g/L deionized water): glucose, 3 ; NaHCO_3_, 2.5; NH_4_Cl, 0.2 ; KH_2_PO_4_, 0.42; KCl, 0.33; NaCl, 0.3; K_2_HPO_4_, 1.26; CaCl_2_.2H_2_O, 0.15; MgCl_2_, 3.15; yeast extract, 1. Ten mL of mineral media were added to the previous constituents. The mineral media consists of (g/L deionized water): EDTA, 0.5; CoCl_2_.6H_2_O, 0.082; CaCl_2_.2H_2_O, 0.114; H_3_BO_3_, 0.01; Na_2_MoO_4_.2H_2_O 0.02; Na_2_SeO_3_, 0.001; Na_2_WO_4_.2H_2_O, 0.01; NiCl_2_.6H_2_O, 0.02; MgCl_2_, 1.16; MnCl_2_.4H_2_O, 0.59; ZnCl_2_, 0.05; CuSO_4_.5H_2_O, 0.01; AlK(SO_4_)_2_, 0.01. The pH value was adjusted at 7 using NaOH solution. The pure six bacterial isolates were reinoculated in sterile nutrient broth separately and incubated for 24 h at 37°C [20], a standard fresh inoculum (10^7^ CFU/mL) of each bacterial isolate was prepared, cultures’ inocula were distributed in the separate chambers of MFCs, and electricity generation was measured every 24 h during a period of five days.

### Molecular identification of the most potent bacterial isolates by 16S RNA

The bacterial isolates were cultured on 5 mL brain heart infusion broth at 37^°^C for 24 h, the grown cells were collected by centrifugation at 12.000 rpm for 2 mins for DNA extraction. The extracted DNA was then used as a template for 16S rRNA gene amplification. through polymerase chain reaction (PCR) using Maxima Hot Start PCR Master Mix (Fermentas). The universal primers used for the amplification were F: 5‘AGAGTTTGATCCTGGCTCAG’3 & R: 5‘GGCTACCTTGTTACGACTT’3. The PCR reaction was performed according to the recommended thermal cycling conditions as follows: denaturation at 95°C for 5 mins (1 cycle), denaturation at 95°C for 30 sec, annealing for at 56°C 30 sec, extension at 72°C for 30 sec, and (40 cycles), and final extension at 72°C for 10 min (1 cycle). Following that, the PCR products were loaded onto a 1.5 percent agarose gel (Biometra, Germany) and electrophoresed in 1 X TBE solution at 100V for approximately 30 mins, it was then dyed with 2µl of 10mg/ml ethidium bromide (Sigma, USA). UV transilluminator (UVP-dual-Intensity Transilluminator,

MultiDoc-It™ system) with wavelength 312nm was used to visualize DNA samples, and a UVP-gel documentation system (DigiCam 130) was used to picture them. Sequencing was performed using the ABI 3730xl DNA sequencer following the manufacturer’s instructions. The sequence was submitted to the database of the National Center for Biotechnology Information (NCBI) GenBank (http://www.ncbi.nlm.nih.gov/BLAST) and compared to other available sequences, and a phylogenetic tree was constructed by the MEGA software version 6 [25].

### Optimization of bacterial growth conditions

Triplicate screw capped 250 mL bottles containing 100 mL of the prepared BM were adjusted at different pH values (6, 6.5, 7, 7.5, 8,) using 1 mol/L HCl and NaOH solutions. The media were inoculated with the selected electrogenic bacterial isolates individually (10^7^ cfu/mL), after 48 h of incubation at 37°C, 100 µL from each bottle was spread on nutrient agar plates, the TVC was recorded (cfu/mL) after 24h of incubation at 37°C and the optimum pH was recorded. For temperature adjustment, triplicate screw capped 250mL bottles containing 100 ml of the prepared BM adjusted at the optimum pH were inoculated (10^7^ cfu/mL), after 48h of incubation at different temperatures (20ºC, 25ºC, 30ºC, 35ºC, 40ºC) the TVC results were recorded as reported before and the optimum temperature was detected. The impact of various substrates on the growth of each electrogenic bacterial isolate was evaluated as the previous experiment by replacing the electron donner (Glucose) with the same concentration of different substrates; Lactose, Fructose and Pyruvate individually and in mixtures, growth results were recorded as cfu/mL, and the optimum substrate was determined [26].

### Assessment of electrogenic potentiality of the selected bacterial isolates individually and in consortium at optimum conditions

MFCs were constructed, stuffed with 500 mL BM medium supplemented with 1% Glucose/Pyruvate as substrate, the optimum growth conditions were maintained before seeding with a standard bacterial inoculum of the two most potent electrogenic bacterial isolates individually and in consortium (10^7^cfu/mL). Electricity generation was measured in terms of output potential difference (mV) with a multi-meter along twelve days with regular intervals. Every 3 days interval 100 mL of 1% substrate was added in the cell after discarding similar volume, to ensure continuous cell operation [27].

### Photo stimulation of electrogenic bacterial consortium

Photo stimulation of the selected electrogenic bacterial consortium was carried out in the National Research Centre (NRC), Egypt using low level red laser (LLRL) tube (ThORLABS, USA) at wave length of 632.8 nm and output power 8 mW, an adjustable stand was used to stabilize the laser tube at 20mm height, irradiation takes place in dark room at 30 ± 2ºC, the bacterial suspension of the bacterial consortium at optimum conditions was subjected to different radiation doses via different exposure intervals (0, 30, 60, 90, 120, 150, 180, 210, 240 sec), the standard bacterial suspensions were inoculated in triplicate screw capped 250mL bottles containing 100mL BM medium, after 24h of incubation at 30ºC the TVC was recorded as cfu/mL as in the optimization step [28].

### Operation of natural sludge based MFC using photo-stimulated bacterial consortium

MFCs were constructed, stuffed with 500 mL sludge diluted with PBS at the ratio of 4:1, the optimum growth conditions were maintained before seeding with a standard inoculum (10^7^cfu/mL) of the irradiated and non-irradiated bacterial consortium individually, Electricity generation was measured in terms of potential difference (mV) with a multi-meter along twelve days with regular intervals. Every 3 days interval 100 mL of 1% substrate was added in the cell after discarding similar volume, to ensure continuous cell operation [27].

### Statistical analysis

Recorded data were analyzed using the statistical package for social sciences, version 23.0 (SPSS Inc., Chicago, Illinois, USA). The quantitative data were presented as mean ± standard deviation and ranges, also qualitative variables were presented as numbers and percentages. A one-way analysis of variance (ANOVA) when comparing between more than two means & Post Hoc test: Tukey’s test was used for multiple comparisons between different variables. Repeated measures analysis of variance (ANOVA) was used to compare multiple within-group measures. The Bonferroni correction was used to adjust the p-value for multiple within-group comparisons.

## Results

### Physicochemical parameters of sludge samples

Four sludge samples were obtained along the period from January 2021 till October 2021 (S1-S4) from El-Shiekh Zayed water purification plant, Giza, Egypt. Physicochemical parameters of the collected sludge samples were recorded (Table 1), results revealed the presence of different metals and ions in the four samples with slight fluctuation in the mean concentration values, also the results of measurement of turbidity, total dissolved solids and electric conductivity were recorded, higher results were detected in S3 sample. The two analytical parameters biological oxygen demand (BOD), and chemical oxygen demand (COD) were estimated, our results showed that compared to other samples S3 had higher COD and BOD values and lower COD/BOD ratio (< 2).

**Table 1:** Physicochemical analysis of the collected sludge samples

Analysis type	Unit	Samples			
		S1	S2	S3	S4
Turbidity	NTU	4000	5800	18462	4500
Temperature	°C	22.4	15.5	27.6	25.2
pH	-	6.9	7.4	7.3	7.0
TDS	mg/l	310	320	340	308
Electric conductivity	Us/cm	470	512	530	465
Total alkalinity	mg/l	612	480	130	530
Total hardness	mg/l	520	540	150	350
Permanent hardness	mg/l	92	60	20	50
Temporary hardness	mg/l	520	480	130	358
Calcium hardness	mg/l	416	300	102	250
Magnesium hardness	mg/l	104	240	48	180
F	mg/l	2.0	0.3	2.5	0.2
SO_4_^2^	mg/l	40.0	39.0	50.0	35.0
PO_4_	mg/l	0.025	19	5.98	18.0
NO_2_	mg/l	0.002	0.01	0.05	0.01
NO_3_	mg/l	UDL	UDL	UDL	UDL
Ca	PPM	166.0	120.0	40.8	118.0
Mg	PPM	25	57.6	11.52	55.0
Al	PPM	0.31	3	0.62	0.55
Fe	PPM	0.18	8.03	2.8	0.36
Li	PPM	0.004	0.20	0.426	0.005
B	PPM	2.899	1.8	4.295	2.0
Na	PPM	143.042	122.0	106.124	111.0
K	PPM	1.015	5.0	6.914	4.0
Ti	PPM	2.474	8.0	19.640	2.1
Cr	PPM	0.099	1.2	0.472	0.9
Mn	PPM	0.306	1.3	18.163	0.5
Co	PPM	0.003	1.5	6.562	1.8
Ni	PPM	0.023	1.3	0.480	1.5
Cu	PPM	0.013	5.2	11.086	2.2
Zn	PPM	0.336	4.4	7.839	4.2
Ga	PPM	0.006	0.01	0.633	0.5
Sr	PPM	0.079	0.6	6.914	0.2
Ag	PPM	0.004	1.2	6.968	0.8
Cd	PPM	0.000	1.3	9.640	0.9
In	PPM	0.000	0.1	0.472	0.1
I	PPM	1.263	2.0	8.163	1.9
Ba	PPM	0.186	1.0	2.649	0.9
Hg	PPM	0.017	0.11	0.562	0.2
Pb	PPM	0.009	0.02	0.480	0.1
Bi	PPM	0.008	0.3	1.086	0.01
BOD	PPM	56.0	55.0	110	35.0
COD	PPM	120	112.0	198	100.0
COD/BOD	-	2.1	2.03	1.8	2.8

### Enumeration of total bacterial counts

**Fig. 2:**
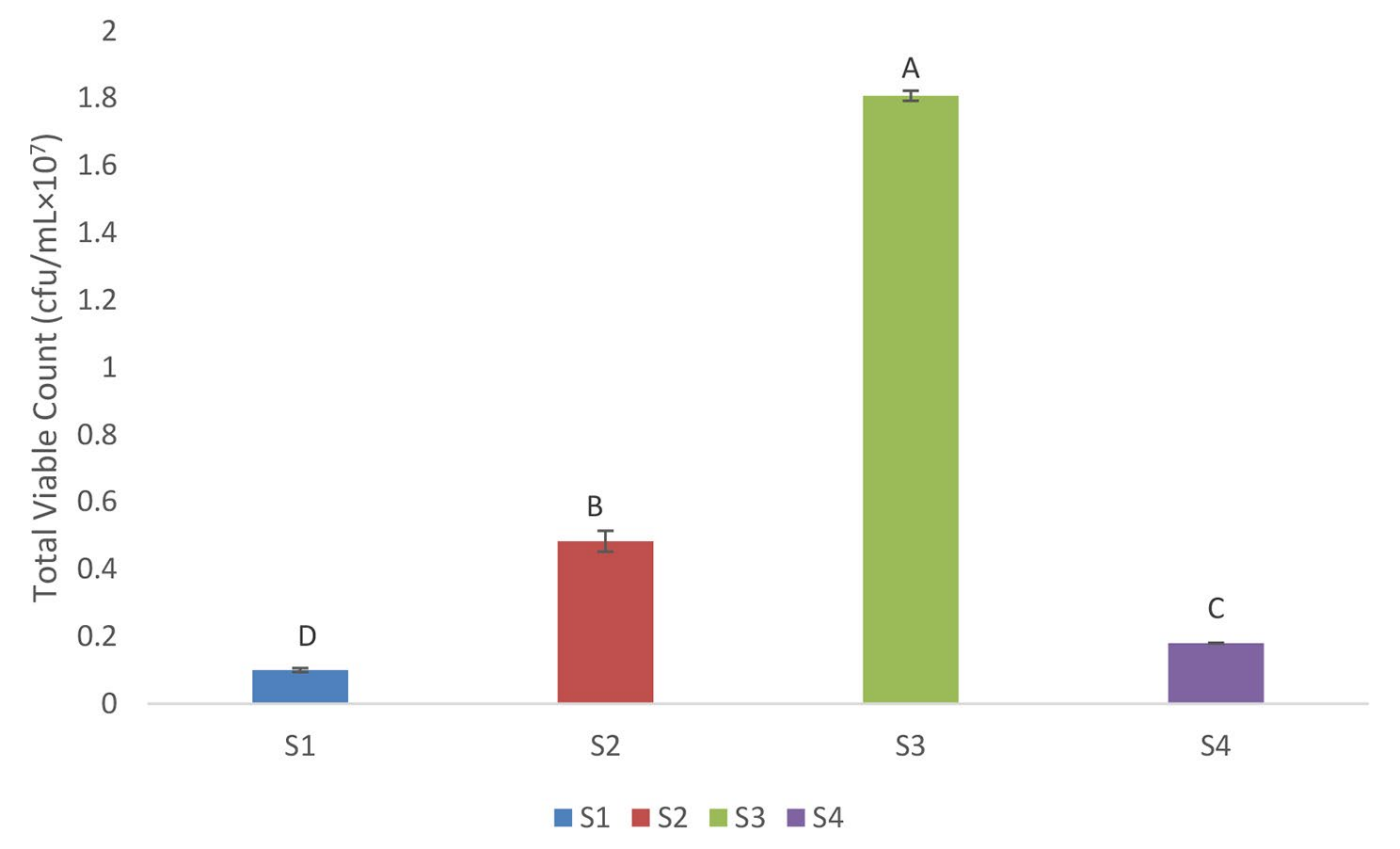
Total viable bacterial count (cfu/mL) in the collected sludge samples. One way Analysis of Variance test was performed for Mean ± SD & Multiple comparison between groups through Post Hoc test: Tukey’s test Letters A-D comparatively represent the descending order of results reflecting importance among means. p-value > 0.05 is insignificant; *p-value < 0.05 is significant; **p-value < 0.001 is highly significant

The mean numerical values of total viable bacterial counts were detected for the four collected sludge samples, the results represented in Fig. 2 showed highly statistically significant value of total viable count (1.8×10^7^ cfu/mL) in S3 sample (p-value < 0.001), while the lowest value was detected in S1 sample (0.1×10^7^ cfu/mL).

#### Detection of electrogenic bacteria from the collected sludge samples

To detect the presence of electrogenic bacteria in the collected sludge samples, single chambered sludge based MFCs were fabricated, the potential differences were measured along 10 days of operation (Table 2), the results indicated that the maximum measured output voltage was ranged from 281.93 to 430.80 mV. For the four samples the electricity generation passed through four stages, stage one which represents the rapid fall in the output voltage, stage two the output voltage remains nearly stable, stage three at which there was a rapid fall in the output voltage, finally stage four which represented a nearly steady state of low voltage.

**Table 2:** Output potential difference values in the collected sludge samples

Time (Days)		Output Potential difference values (mV)				F-test	p-value
		S1	S2	S3	S4		
Day 0	Stage One	281.93 ± 0.83D	365.23 ± 0.25B	430.80 ± 0.98A	297.00 ± 0.50C	49.539	< 0.001**
Day 1		177.87 ± 0.78D	262.50 ± 0.50B	310.90 ± 0.66A	189.50 ± 0.50C	53.756	< 0.001**
Day 2	Stage Two	150.80 ± 0.62C	200.60 ± 0.66B	270.53 ± 0.45A	156.70 ± 0.44C	68.468	< 0.001**
Day 3		146.83 ± 0.47C	198.50 ± 0.50B	250.67 ± 0.40A	151.90 ± 0.26A	23.658	< 0.001**
Day 4		133.80 ± 0.62D	182.00 ± 0.90B	211.00 ± 0.20A	147.87 ± 0.51C	33.974	< 0.001**
Day 5	Stage Three	84.43 ± 0.31D	105.93 ± 0.60B	136.23 ± 0.25A	92.63 ± 0.45C	20.381	< 0.001**
Day 6		63.30 ± 0.26D	75.37 ± 0.47B	98.37 ± 0.25A	71.53 ± 0.40C	49.050	< 0.001**
Day 7		31.33 ± 0.15D	43.70 ± 0.44B	50.70 ± 0.46A	38.67 ± 0.35C	79.031	< 0.001**
Day 8	Stage Four	28.17 ± 0.15D	41.83 ± 0.76B	48.67 ± 0.50A	34.77 ± 0.32C	20.399	< 0.001**
Day 9		26.23 ± 0.25D	37.47 ± 0.45B	46.40 ± 0.26A	31.63 ± 0.35C	35.520	< 0.001**
Day 10		24.30 ± 0.20D	35.53 ± 0.55A	30.23 ± 26.19A	29.27 ± 0.25C	33.627	< 0.001**

Data are expressed by Mean ± SD & Multiple comparison between groups through Post Hoc test: Tukey’s test.

Letters A-D comparatively represent the descending order of results reflecting importance among means in the same row p-value > 0.05 is insignificant; *p-value < 0.05 is significant; **p-value < 0.001 is highly significant

### Isolation and primary identification of bacterial strains

Six distinct bacterial isolates were isolated from S3 sludge sample, the bacterial isolates were purified and primary identified using VITEK®2 system which permits rapid identification and antimicrobial susceptibility testing of the bacteria, the results illustrated in Table 3 showed that the six isolates were identified as *Aeromonas salmonicida* (*A. salmonicida*), *Pantoea sp, Enterobacter cloacae* (*E. cloacae*), *Staphylococcus lentus* (*S. lentus*), *Comamonas testosterone* (*C. testosterone*) and *Escherichia coli* (*E. coli*).

**Table 3:** Identification of the isolated bacterial strains by VITEK® 2 System

Test	Mnemonic	*A. salmonicida*	*Pantoea sp.*	*E. cloacae*	*S. lentus*	*C. testosterone*	*E. coli*
Ala-Phe-Pro Arylamidase	APPA	-	-	-	-	-	-
L-Pyrrolidonyl Arylamidase	PyrA	-	-	-	-	+	-
Beta-Galactosidase	BGAL	-	-	+	-	-	+
D-Glucose	dGLU	-	+	+	-	-	+
^γ^ Glutamyl Transferase	GGT	-	-	+	-	-	+
Fermentation/Glucose	OFF	-	-	+	-	-	+
Beta-Glucosidase	BGLU	-	+	-	-	-	+
D-Maltose	dMAL	-	+	+	-	-	+
D-Mannitol	dMAN	-	+	+	+	-	+
D-Mannose	dMNE	-	+	+	+	-	+
Beta-Xylosidase	BXYL	-	-	+	-	-	+
Beta-Alanine arylamidase pDNA	BALap	-	-	-	-	-	+
L-Proline Arylamidase	ProA	+	-	-	-	-	-
Lipase	LIP	+	-	-	-	-	-
Palatinose	PLE	-	-	+	-	-	-
Tyrosine Arylamidase	TyrA	-	-	+	-	+	-
Urease	URE	-	-	-	-	-	-
D-Sorbitol	dSOR	-	-	+	-	-	-
Sucrose	SAC	-	-	+	-	-	+
D-Tagatose	dTAG	-	-	-	-	-	+
D-Trehalose	dTRE	-	+	+	-	-	+
Citrate	CIT	-	-	+	-	-	-
Malonate	MNT	-	+	+	-	-	-
5-Keto-D-Gluconate	5KG	-	-	-	-	-	+
Alpha-Glucosidase	AGLU	-	-	-	-	-	-
Succinate Alkalinization	SUCT	-	-	+	-	+	-
Beta-N-Acetyl-Galactosaminidase	NAGA	-	-	+	+	-	-
Alpha-Galactosidase	AGAL	-	-	+	-	-	-
Phosphatase	PHOS	-	-	-	+	-	+
Glycine Arylamidase	GlyA	-	-	+	-	-	-
Ornithine Decarboxylase	ODC	-	-	+	-	-	+
Lysine Decarboxylase	LDC	-	-	-	-	-	+
Courmarate	CMT	-	-	+	-	-	-
Beta-Glucuronidase	BGUR	-	-	-	-	-	+
Glu-Gly-Arg- Arylamidase	GGAA	-	-	-	-	+	-
Ellman	ELLM	-	-	-	-	+	-

### Screening and molecular identification of the most potent electrogenic bacterial isolates

Synthetic growth medium was prepared to be used as a nutrient supplement to enhance the growth and oxidation power for electrogenic bacteria, results represented in Table 4 revealed that among the six bacterial isolates, five were found to have electrogenic behavior, as no readings were recorded for *Pantoea* sp. *E. cloacae* and *E. coli* were selected to be significantly the most potential based on the measurements of the output potential difference along a period of five days.

**Table 4:** Electrogenic potentials of the isolated bacterial strains

Time (Days)	Potential difference (mV)				
	*A. salmonicida*	*E. cloacae*	*S. lentus*	*C. testosterone*	*E. coli*
One	28.47 ± 0.81C	112.77 ± 0.31A	20.60 ± 0.78E	22.83 ± 0.25D	98.73 ± 0.38B
Two	30.13 ± 1.06C	115.93 ± 0.67A	21.87 ± 0.67D	24.17 ± 0.31D	100.47 ± 0.25B
Three	32.23 ± 1.16C	118.73 ± 0.68A	26.33 ± 0.31D	26.80 ± 0.30D	104.07 ± 0.45B
Four	20.47 ± 0.60C	90.50 ± 0.53A	15.53 ± 0.35E	16.73 ± 0.40D	65.90 ± 0.44B
Five	16.47 ± 0.65C	74.33 ± 0.59A	11.33 ± 0.47E	14.40 ± 0.26D	45.77 ± 0.42B
**RMANOVA**	174.687	3434.438	320.862	938.691	1297.205
**p-value**	< 0.001**	< 0.001**	< 0.001**	< 0.001**	< 0.001**

Repeated-measures analysis of variance (ANOVA) & Multiple comparison between groups through Post Hoc test: Bonferroni-corrected. Letters A-E comparatively represent the descending order of results reflecting importance among means at the same time. p-value > 0.05 is insignificant; *p-value < 0.05 is significant; **p-value < 0.001 is highly significant

### Molecular identification of the most potent bacterial isolates by 16S RNA

**Fig. 3:**
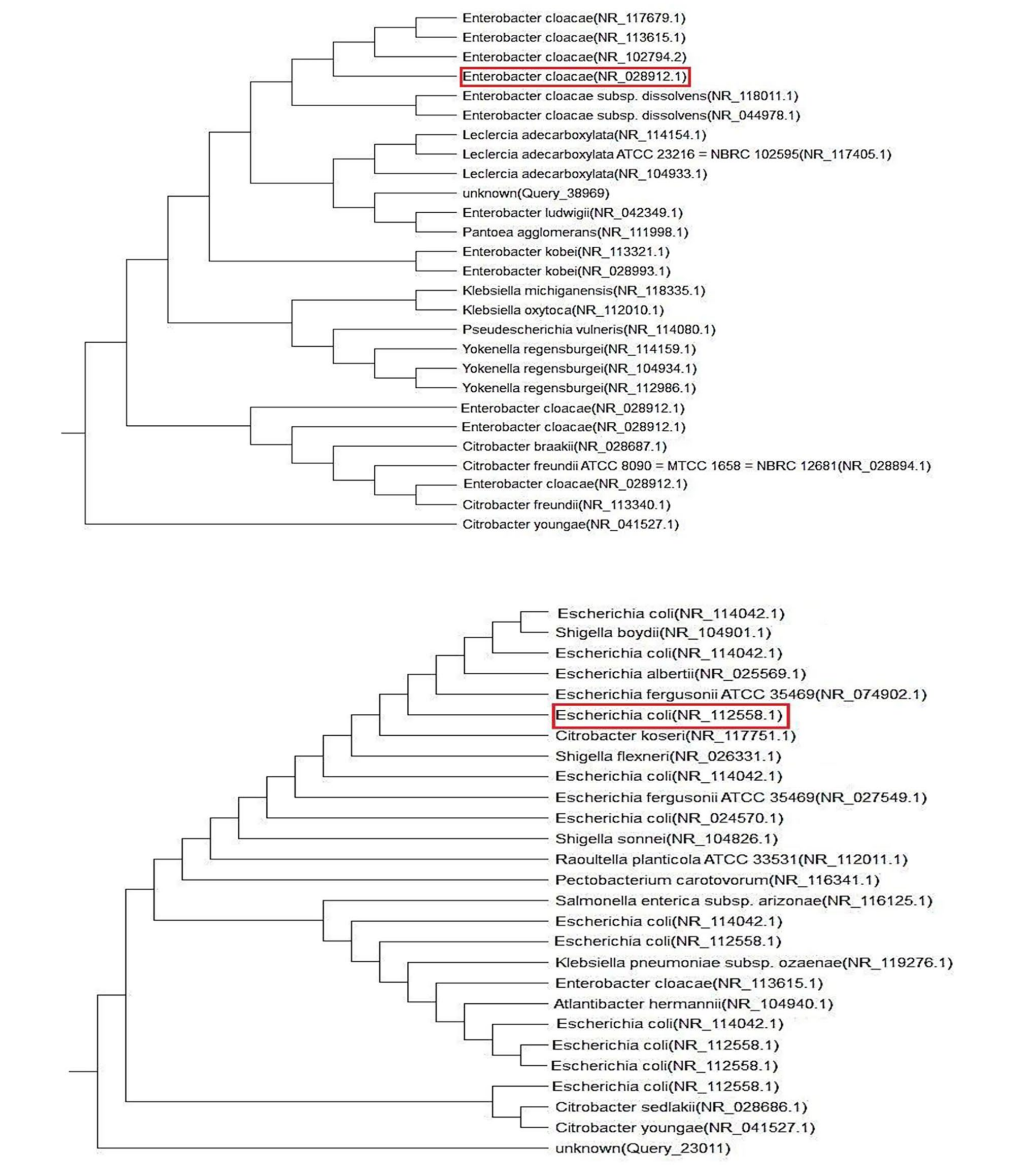
Phylogenetic tree of *E. coli and E. cloacae* based on 16S rRNA constructed using the neighbor joining method.

The most potent electrogenic bacteria (*E. cloacae* and *E. coli*) were subjected to confirmatory molecular characterization by using the 16S ribosomal RNA (16S rRNA) marker gene. The Basic Local Alignment Search Tool (BLAST) homology search and motif analysis revealed that the strains were located in the positions occupied by the genera *Enterobacter* and *Escherichia* Fig. 3 declared the results of the phylogenetic characterization and showed that strains NR_028912.1 and NR_112558.1 were most likely related to the strains *E. cloacae* and *E. coli* respectively.

DNA fragments of genes of the Egyptian strains *E. coli* and *E. cloacae* were subjected to DNA sequencer to reveal their internal structure. Sequence alignment of 16S rRNA genes were done on the GenBank Database on the World Wide Web (see supplementary Fig S1 online), for *E. coli* gene the results illustrated sixty-nine types of mutations including substitution (inversion and transversion mutation), deletion and insertion mutations. Inversion mutations were detected at positions Q20 Q268 Q321 (G > A), Q21 Q47 Q64 Q73 Q82 Q95 Q121 Q131 Q133 Q153 Q252 Q304 Q352 (A > G), Q128 (C > T) and Q237 (T > C). At the same time transversion mutations were recorded at positions Q3 Q69 Q111 Q115 Q266 Q276 Q 290 Q357 (G > T), Q9 Q168 Q205 Q260 Q341 (T > G), Q17 Q52 Q58 Q90 Q107 Q158 Q273 (C > G), Q67 (A > C), Q78 Q137 Q140 Q248 Q340 Q360 (T > A), Q88 Q173 Q185 Q315 (C > A) and Q105 Q174 Q201 (A > T). Deletion mutations were detected at between positions Q34-35 (-T), Q114-115, Q315-316 (-A), Q135-136, Q281-282, Q315-316 (-G) and Q266-267 (-C). At the same time insertion mutations were recorded at positions Q38 Q295 (+ T), Q144 (+ G), Q279 Q301 Q328 (+ A) and Q323 Q324 Q325 Q353 (+ C). For *E. cloacae* there was one type of mutation including substitution mutation (inversion mutation) which was detected at position Q802 (C > T) (see supplementary Table S2 online).

### Optimization of the growth conditions of the most potent electrogenic bacteria

**Fig. 4:**
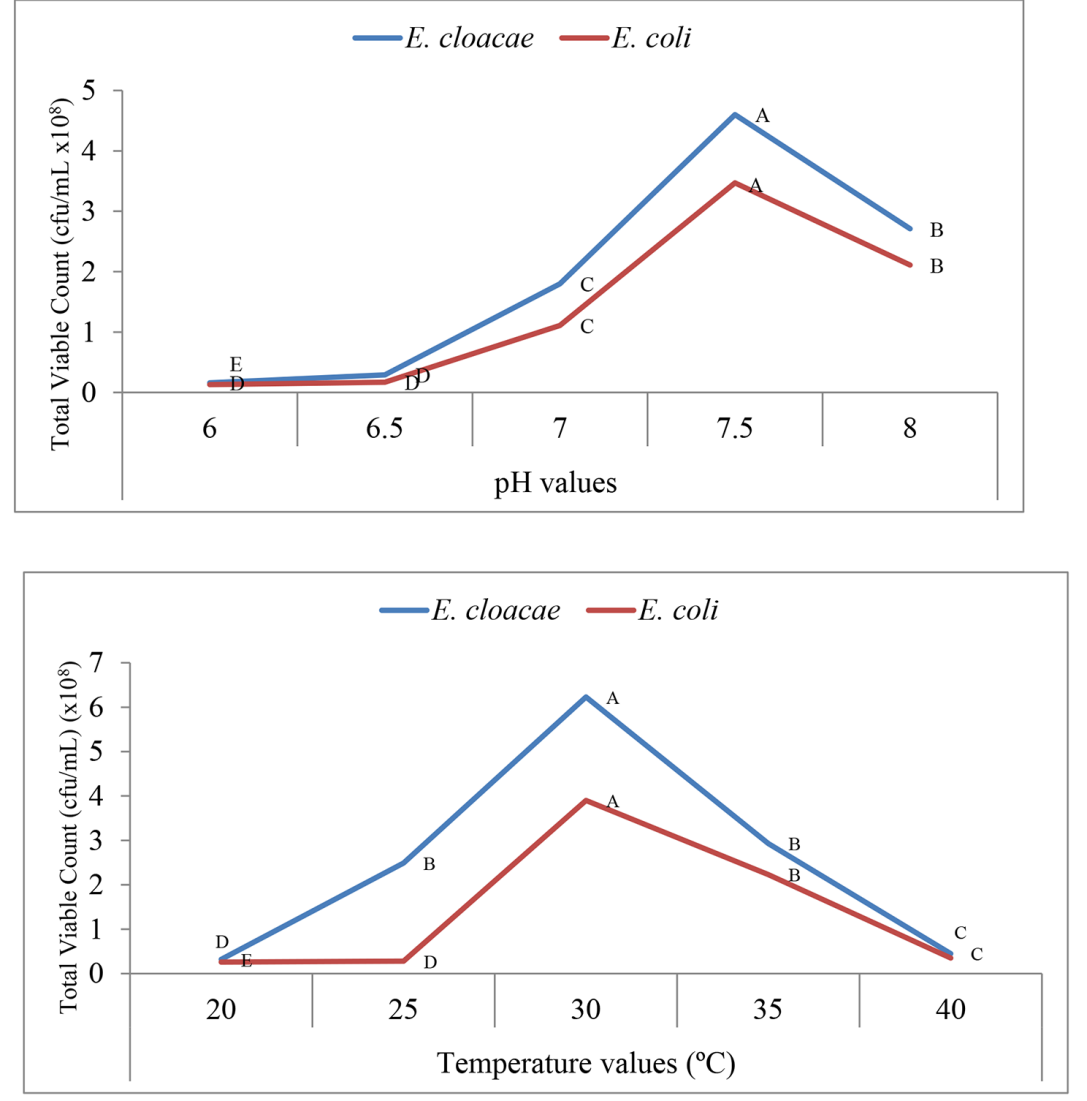
Total viable count (cfu/mL) at different ph and temperature values. Repeated-measures analysis of variance (ANOVA) & Multiple comparison between groups through Post Hoc test: Bonferroni-corrected. Letters A- E comparatively represent the descending order of results reflecting importance among means at the same value, p-value > 0.05 is insignificant; *p-value < 0.05 is significant; **p-value < 0.001 is highly significant

As shown in Fig. 4 the statistically significant higher mean values of total viable counts (cfu/mL) were recorded at pH 7.5 and temperature 30°C for both *E. cloacae* and *E. coli* with p-value (p < 0.001) so they are considered the most optimum and their values are taken for further studies.

**Fig. 5:**
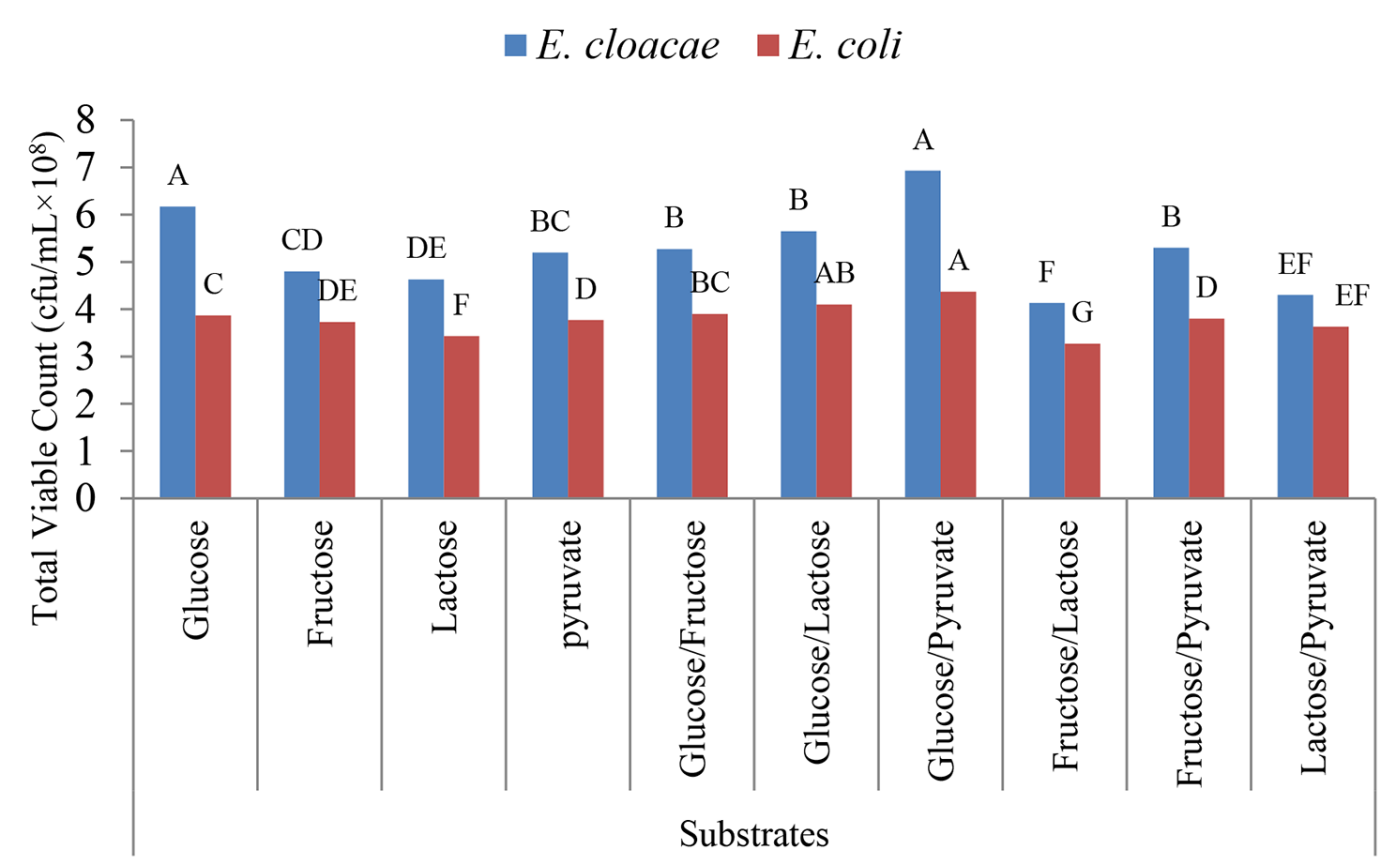
Total viable count (cfu/mL) using different types of substrates. Repeated-measures analysis of variance (ANOVA) & Multiple comparison between groups through Post Hoc test: Bonferroni-corrected. Letters A-F comparatively represent the descending order of results reflecting importance among means at the same value, p-value > 0.05 is insignificant; *p-value < 0.05 is significant; **p-value < 0.001 is highly significant

The results represented in Fig. 5 revealed statistically significant higher mean value of total viable counts (cfu/mL) for *E. cloacae* and *E. coli* were recorded on using glucose/ pyruvate with p-value (p < 0.001).

### Electrogenic potentiality of the selected bacterial isolates individually and in consortium at optimum conditions

**Fig. 6:**
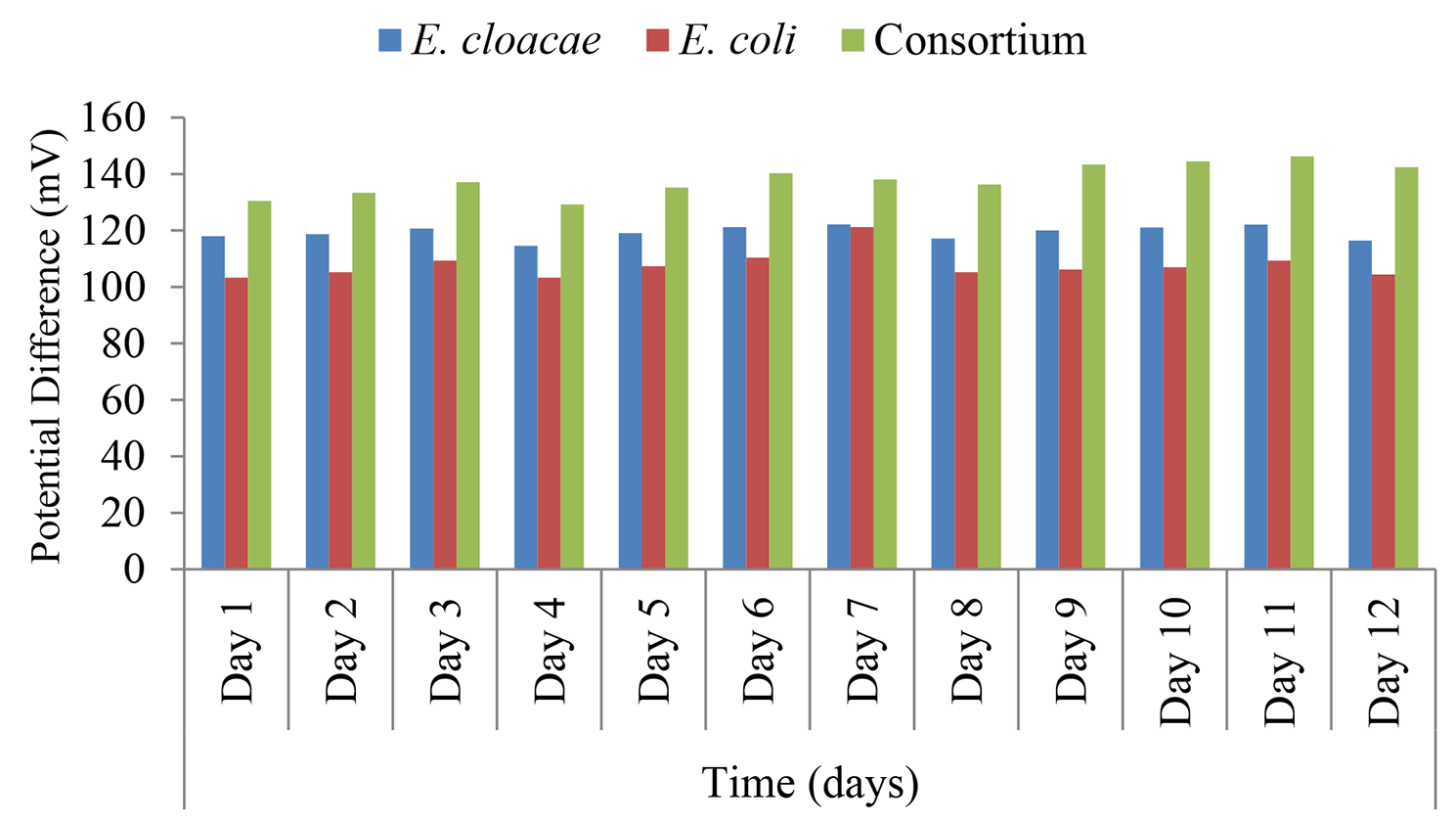
Pattern of potential difference output for *E. cloacae, E. coli* and their consortium. Paired Sample t-test. p-value > 0.05 is insignificant; *p-value < 0.05 is significant; **p-value < 0.001 is highly significant

MFC loaded with synthetic media at optimum conditions was used to find out the electrogenic potentiality of *E. cloacae* and *E. coli* individually and in consortium, electricity generation in terms of potential difference (mV) was recorded using multimeter from day one and extends throughout twelve days, the results represented in Fig. 6 declared that for both isolates and their consortium the electricity generation was in direct proportion with time till day three at which the recorded values started to decrease due to depletion of the substrate, on supplying the MFC with 1% Glucose/Pyruvate at regular intervals, the mean values of potential difference started to rise, the results showed statistically significant higher mean value of Potential difference (mV) in bacterial consortium compared to both isolates individually at each time (p < 0.001).

### Photo stimulation of electrogenic bacterial consortium

**Fig. 7:**
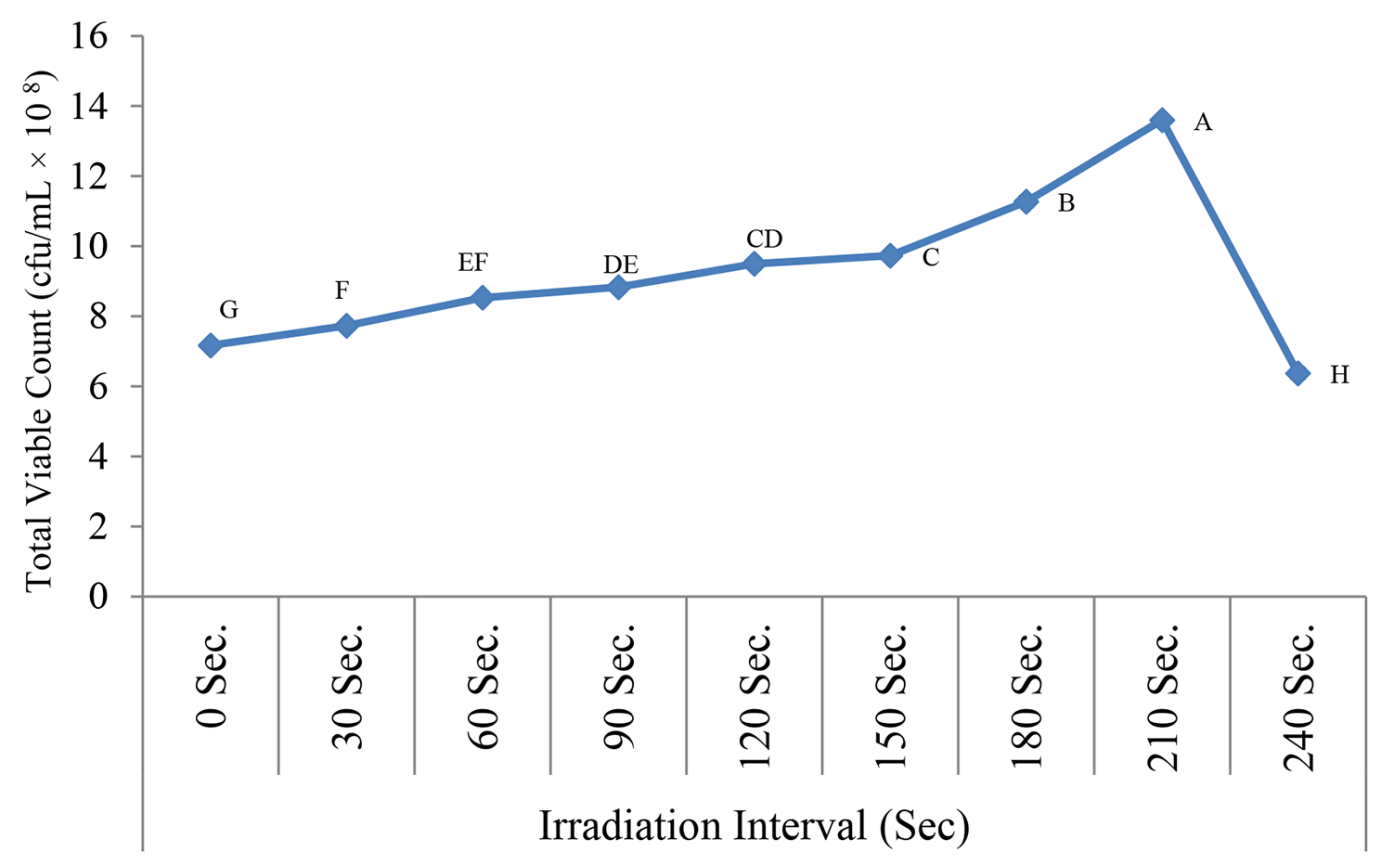
Effect of LLRL on the bacterial growth of the electrogenic bacterial consortium. Repeated-measures analysis of variance (ANOVA) & Multiple comparison between groups through Post Hoc test: Bonferroni-corrected, letters A-H comparatively represent the descending order of results reflecting importance among means at the same value. p-value > 0.05 is insignificant; *p-value < 0.05 is significant; **p-value < 0.001 is highly significant

Our results clarified a dose dependent stimulatory effect of LLRL on the growth rate of the bacterial consortium (Fig. 7), significant maximum population was recorded at 210 sec of irradiation (13.5 × 10^8^ cfu/mL value (p < 0.001), on the other hand increasing the irradiation interval resulted in photoinhibition leading to significant decrease in the TVC.

### Operation of natural sludge based MFC using photo-stimulated bacterial consortium

**Fig. 8:**
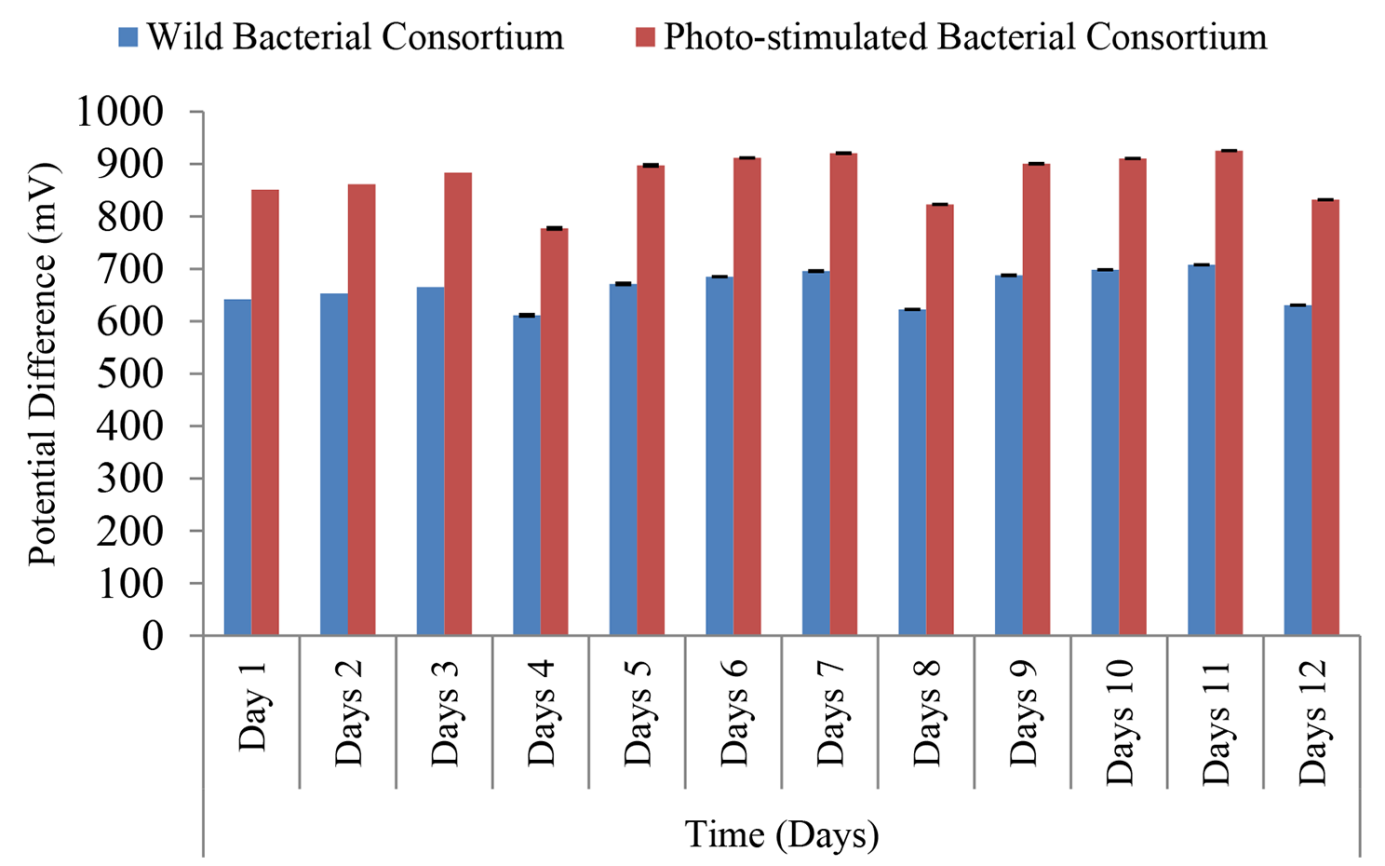
Pattern of potential difference using photo-stimulated bacterial consortium. Paired Sample t-test. p-value > 0.05 is insignificant; *p-value < 0.05 is significant; **p-value < 0.001 is highly significant

Sludge based MFC was constructed as a kind of application to use sludge obtained from chemical precipitation, sedimentation or any other process in the generation of green electricity, our results (Fig. 8) declared that addition of exogenous bacterial inoculum of wild and photo-stimulated bacterial consortium showed high synergistic action, leading to increasing the performance of the MFC, there was a statistically significant higher mean value of potential difference (mV) in photo-stimulated bacterial consortium compared to wild bacterial consortium in each time (p < 0.001), the addition of fresh medium containing glucose/pyruvate (1%) at regular intervals over comes the depletion of nutrients and led to the revival of the endogenous and exogenous microbial flora, thus the mean value of the potential difference was maintained during the time of operation.

## Discussion

Sludge production volumes have been increased steadily every year [29]. Major issues arise when large amounts of sludge are dumped into the environment, including worsened odors, an increased risk of pathogenic microorganisms, and heavy metal accumulation [30]. In the present study four sludge samples acting as a microbial source were obtained from El-Shiekh Zayed water purification plant, the higher recorded results of the concentrations of metals and ions detected in S3 sample might be due to the rain effect which causes soil erosion, so different elements were then transported to the combined sanitation network and dumped into the water treatment plant as described by Brahimi et al. [31], also the higher values of turbidity and electric conductivity reflects higher microbial load as reported by Mucha and Kułakows [32]. The two analytical parameters biological oxygen demand (BOD), and chemical oxygen demand (COD) reflected the oxygen content possibly present due to pollutants in the investigated sludge samples, BOD determines the amount of dissolved oxygen consumed by bacteria to oxidize organic compounds, while COD is an indicative measure of the amount of oxygen needed for complete oxidation of organic compounds [33], higher BOD and COD values reveled higher concentration of organic substrates needed for the persistent microflora, additionally low COD/BOD ratio (< 2) indicated high performance of oxidation-reduction reactions by the naturally occurring microflora [34]. Temperature affected the physicochemical parameters of sludge samples, so it is considered as a key factor in shaping the structures of microbial populations. High records of microbial loads of S3 sample are harmonious with the results of Madni et al [35] who reported that high temperature favors the survival of microorganisms. The global energy crisis and the limited resources necessitates the search for alternative sustainable energy sources. in MFCs the organic matter present in the sludge samples was subjected to biochemical conversion system to produce electric current [36], the electrogenic microflora acts as a catalyst that oxidizes the organic matter resulting in the release of electrons and protons, electrons were migrated to the anode and consequently transferred to the cathode through an external wire, while the protons transferred directly to the cathode through the solution, the created potential difference can be recorded [37]. The measured values of potential difference (mV) indicated that the microflora in all samples have electrogenic potential and they can manifest this potential if they are properly re-inoculated into their habitat, the electricity generation passes through four stages initiated by a rapid fall in the output voltage, then a nearly stable interval of electricity generation, followed by another rapid fall in the output voltage, finally a stage of nearly steady state of low voltage. Similar electricity generation pattern was recorded by Cau et al [2], the highly statistically significant results recorded for S3 samples are in correspondence to that of the TVC results. The isolation of six bacterial strains from the selected sludge sample declared the fact that electrogenic bacteria are present in all ecosystems [38], with different electrogenic potentialities due to their multiple reducing mechanisms [39], primary and rapid identification using VITEK®2 system showed that our isolated bacterial collection included *A. salmonicida* which is associated with fish [40], *S. lentus, E. cloacae*, and *E. coli* which are associated with animals [41], *C. testosterone* and *Pantoea sp* which are mainly found in soil, waste water and sludge [42], in agreement with the results recorded by Nguyena et al [43] the most potent electrogenic isolates were *E. cloacae*, and *E. coli*, consequently they were subjected to confirmatory molecular characterization by using the 16S rRNA marker gene which has served as an important tool for determining phylogenetic relationships between bacteria. [44]. BLAST was applied to find regions of similarity between biological sequences and the results of phylogenic characterization showed that the strains NR_028912.1 and NR_112558.1 were most likely related to the strains *E. cloacae* and *E. coli* respectively, the sequence alignment of 16S rRNA gene illustrated the presence of some mutations including substitution (inversion and transversion mutation), deletion and insertion in both isolates. Generation of green electricity depends on the massive growth of electrogenic bacteria which carry out oxidation reduction reactions to generate electric current, growth conditions play a significant role in the biomass production of the microorganisms that are capable of converting pollutants to electric current via electrochemical reactions [45]. There are many physical, chemical and biological parameters that affect the operation of MFCs [46]. In our study the optimum conditions studied for isolated electrogenic bacterial isolates (*E. cloacae* and *E. coli*) were evaluated by determination of the total viable bacterial count in terms of cfu/mL as their increase reflects the ability of substrate utilization and consequently increasing electrons flow rate [26]. pH value is one of the crucial factors that significantly impact the bacterial growth rate, it affects the catalytic ability of enzymes, and it also alters the ionic charge of many biomolecules affecting the metabolic reactions [47]. for both *E. cloacae* and *E. coli* was pH 7.5 was reported as the optimum pH value at which maximum total viable count was achieved. Near results were obtained by Kim et al. [17] who reported that the optimum pH range for the growth of Enterobacteriaceae was between 6 and 8. Temperature affects the spatial arrangement of the atoms in the biomolecules and hence at the optimum temperature the biomolecules retain a stable and active structure which enables it to carry out its function. At low temperatures, enzymes stop functioning as low temperatures increase the viscosity of fluids and hardening of lipids, while high temperatures cause rupture of hydrogen bonds in proteins and in DNA resulting in protein denaturation [48]. It was evident from our results that the optimum incubation temperature at which the maximum population was 30°C. These results are consistent with those reported by Sihag et al [49] who indicated that most bacteria are active in the mesophilic range of 25°C to 40°C. Nutrient utilization strategy affect the growth rate of microbial cells, microorganisms can grow on a variety of growth substrates and hence possess versatile metabolic activities, previous literature reported that carbon sources are categorized into groups based on their mechanism of joining the metabolic pathway [48], in our study higher population scores were recorded on using glucose/pyruvate, these results are consistent with those of Wang et al [50] who reported that the bacterial isolates tend to co-utilize both sources simultaneously leading to catabolite motivation. It is well known that the efficient applicability of MFC systems depends on the enzymatic degradation ability of the electrogenic microorganisms, to overcome this challenge mixed cultures were suggested [51], so using the two most potent electrogenic bacterial isolates (*E. cloacae* and *E. coli*) in consortium was significantly more efficient than using them individually. In agreement with the findings of Khater et al [22] who reported that, regular addition of fresh substrate overcome the depletion of nutrients and activate the bacterial culture leading to the restoration of the cell potential, feeding the MFC with 100mL glucose/pyruvate at regular intervals maintain a steady energy generation along the interval of operation. Trying to improve the performance of MFCs, the bacterial consortium was subjected to LLRL to enhance the cell proliferation, protein synthesis and metabolic activities [20]. Previous studies on the effect of laser radiation indicates its bio-stimulatory effect, this effect returned to the photoexcitation of the protein cytochrome C complex, consequently it pumps more photons thus the amount of ATP increases leading to increase in the growth rate, on the other hand high irradiation doses causes cytotoxic effect leading to the damage of the cell structure [28], our results are harmonious with those of Crugeira et al [18] who reported that the stimulatory effect of LLRL is dose dependent and has positive influence on thermophilic bacterial kinetics. In an application of assessing the efficiency of new MFC designed using sludge which contain the metabolically active bacterial cells as culture medium was done, addition of exogenous bacterial inoculum of the photo-stimulated bacterial consortium led to significantly high synergistic action that increased the cell performance, as acceleration of cell proliferation consequently led to intensifying the electron flow, also the addition of fresh medium containing glucose /pyruvate (1%) at regular intervals over comes the depletion of nutrients and led to the revival of the endogenous and exogenous microbial flora, thus the mean value of the potential difference was maintained during the time of operation.

## Conclusion

The main objective of this work was to introduce an ecofriendly and economically viable method to generate green electricity, promising results were obtained by utilization of the potent electrogenic bacterial strains *E. coli* and *E. cloacae* as consortium at optimum conditions. Low level red laser was applied to boost the electrogenic potentiality of the bacterial consortium, results declared significant increase in the efficiency of MFC inoculated with the photo-stimulated consortium. The approach of using photo-stimulated bacterial strains serves as a dual solution for both pollution treatment and renewable energy generation. In future work, in depth study of the enzymatic mechanism of the dual role of photo-stimulated electrogenic bacterial consortium in renewable energy and biohydrogen production will be performed.

### Electronic Supplementary Material

Below is the link to the electronic supplementary material

Supplementary Material 1

## Data Availability

The datasets used or analyzed during the current study are available from the corresponding author on reasonable request.
