# Supplementary material for: Generation of green electricity from sludge using photo-stimulated bacterial consortium as a sustainable technology
Source: Microb Cell Fact. 2023 Sep 15;22:183. doi: 10.1186/s12934-023-02187-y (PMC10503168; doi:10.1186/s12934-023-02187-y)
Supplement: Supplementary file 1 — Supplementary Material 1 [file 12934_2023_2187_MOESM1_ESM.docx]

**Supplementary Figure S1. Sequence Alignment of 16S rRNA Gene of *E. coli* and *E. cloacae* Compared to The Genbank.**


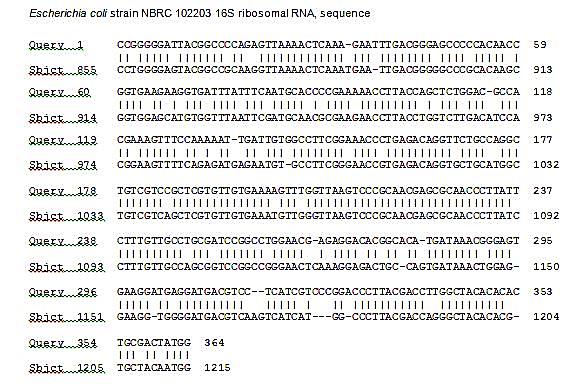


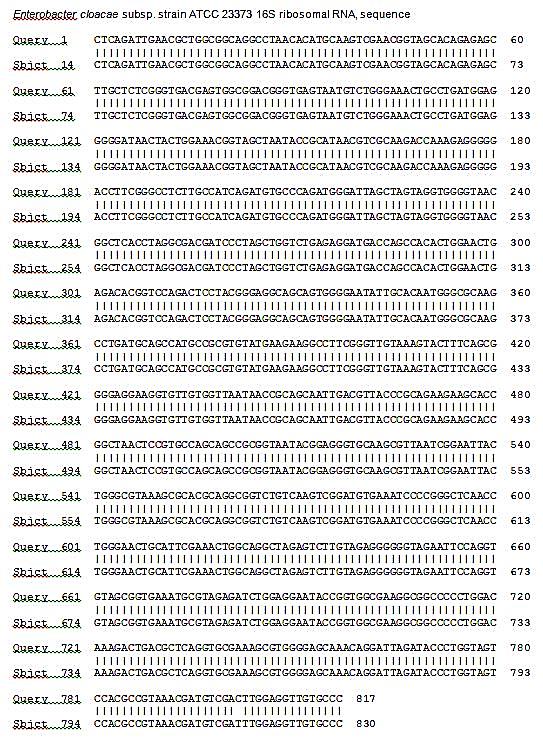


**Supplementary Table S2. Types of Mutations Detected in Complex Region of 16S rRNA Genes of *E. coli* and *E. coloacae* Egyptian Isolates Compared to The Same Region Recorded in GenBank Data Base.**

| **Position** | **Type of Mutation** | | | |
| --- | --- | --- | --- | --- |
| **(Query)** | **Deletion** | **Substitution** | | **Insertion** |
|  |  | **Inversion** | **Transversion** |  |
| ***E. coli*** | | | | |
| **3** | -- | -- | G > T | -- |
| **9** | -- | -- | T > G | -- |
| **17** | -- | -- | C > G | -- |
| **20** | -- | G > A | -- | -- |
| **21** | -- | A > G | -- | -- |
| **34 – 35** | -T | -- | -- | -- |
| **38** | -- | -- | -- | +T |
| **47** | -- | A > G | -- | -- |
| **52** | -- | -- | C > G | -- |
| **58** | -- | -- | C > G | -- |
| **64** | -- | A > G | -- | -- |
| **67** | -- | -- | A > C | -- |
| **69** | -- | -- | G > T | -- |
| **73** | -- | A > G | -- | -- |
| **78** | -- | -- | T > A | -- |
| **82** | -- | A > G | -- | -- |
| **88** | -- | -- | C > A | -- |
| **90** | -- | -- | C > G | -- |
| **95** | -- | A > G | -- | -- |
| **105** | -- | -- | A > T | -- |
| **107** | -- | -- | C > G | -- |
| **111** | -- | -- | G > T | -- |
| **114 – 115** | -A | -- | -- | -- |
| **115** | -- | -- | G > T | -- |
| **121** | -- | A > G | -- | -- |
| **128** | -- | C > T | -- | -- |
| **131** | -- | A > G | -- | -- |
| **133** | -- | A > G | -- | -- |
| **135 – 136** | -G | -- | -- | -- |
| **137** | -- | -- | T > A | -- |
| **140** | -- | -- | T > A | -- |
| **144** | -- | -- | -- | +G |
| **153** | -- | A > G | -- | -- |
| **158** | -- | -- | C > G | -- |
| **168** | -- | -- | T > G | -- |
| **173** | -- | -- | C > A | -- |
| **174** | -- | -- | A > T | -- |
| **185** | -- | -- | C > A | -- |
| **201** | -- | -- | A > T | -- |
| **205** | -- | -- | T > G | -- |
| **237** | -- | T > C | -- | -- |
| **248** | -- | -- | T > A | -- |
| **252** | -- | A > G | -- | -- |
| **260** | -- | -- | T > G | -- |
| **266** | -- | -- | G > T | -- |
| **266 – 267** | -C | -- | -- | -- |
| **268** | -- | G > A | -- | -- |
| **273** | -- | -- | C > G | -- |
| **276** | -- | -- | G > T | -- |
| **279** | -- | -- | -- | +A |
| **281 – 282** | -G | -- | -- | -- |
| **290** | -- | -- | G > T | -- |
| **295** | -- | -- | -- | +T |
| **301** | -- | -- | -- | +A |
| **304** | -- | A > G | -- | -- |
| **315** | -- | -- | C > A | -- |
| **315 – 316** | -A -G | -- | -- | -- |
| **321** | -- | G > A | -- | -- |
| **323** | -- | -- | -- | +C |
| **324** | -- | -- | -- | +C |
| **325** | -- | -- | -- | +C |
| **328** | -- | -- | -- | +A |
| **340** | -- | -- | T > A | -- |
| **341** | -- | -- | T > G | -- |
| **352** | -- | A > G | -- | -- |
| **353** | -- | -- | -- | +C |
| **357** | -- | -- | G > T | -- |
| **360** | -- | -- | T > A | -- |
| ***E. cloacae*** | | | | |
| **802** | -- | C > T | -- | -- |
